# Development of Analytical Methods for Determination of *β*-Carotene in Pumpkin (*Cucurbita maxima*) Flesh, Peel, and Seed Powder Samples

**DOI:** 10.1155/2022/9363692

**Published:** 2022-02-11

**Authors:** Mulu Hagos, Mesfin Redi-Abshiro, Bhagwan Singh Chandravanshi, Estifanos Ele Yaya

**Affiliations:** Department of Chemistry, College of Natural and Computation Sciences, Addis Ababa University, P.O. Box 1176, Addis Ababa, Ethiopia

## Abstract

Vegetables are consumed worldwide in fresh as well as processed forms. Pumpkin is considered as an important vegetable due to its nutritional values. The objective of this study was to evaluate all the analytical parameters and improve the performance of the methods for the determination of *β*-carotene in pumpkin flesh, peel, and seed samples using UV-VIS, NIR, and FTIR methods. *β*-Carotene was measured at 453 nm using the UV-VIS method which showed linear range, 0.1 to 12 µg/mL; *R*^2^, 0.999; LOD, 0.034 µg/mL; LOQ, 0.1 µg/mL; RSD, 1.5% to 11%; and percent recovery, 83% to 93%. *β*-Carotene was also measured at 1415 nm using NIR and at 1710 cm^−1^ using FTIR spectroscopic methods. The NIR method exhibited linear range, 12.5 to 250 µg/mL; *R*^2^, 0.999; LOD, 3.4 µg/mL; LOQ, 10, µg/mL; RSD, 1.8% to 11%; and percent recovery, 92% to 96%, while the FTIR method exhibited linear range, 4 to 100 µg/mL; *R*^2^, 0.999; LOD, 1.3 µg/mL; LOQ, 3.9 µg/mL; RSD, 0.69% to 10%; and percent recovery, 95% to 96%. The characteristic analytical data indicated that any of the three newly developed methods could be used for the determination of *β*-carotene in the pumpkin flesh, peel, and seeds. Acetone was used as the extraction solvent for the UV-VIS and NIR spectroscopic methods, and acetonitrile was used as the extraction solvent for the FTIR method. Content of *β*-carotene was found higher in pumpkin peel (340–445 µg/g), followed by pumpkin flesh (317–341 µg/g) and pumpkin seed (12–17 µg/g) by the UV-VIS method. *β*-Carotene content was obtained ((376–451 µg/g) and (289–313 µg/g); (210–287 µg/g) and (102–148 µg/g)) using NIR and FTIR methods in pumpkin peel and flesh, respectively. *β*-Carotene was obtained higher from pumpkin peel by all the three methods than from pumpkin flesh and seed. The *β*-carotene content in the pumpkin seed was not detected by NIR and FTIR spectroscopic methods.

## 1. Introduction

**Figure 1:**
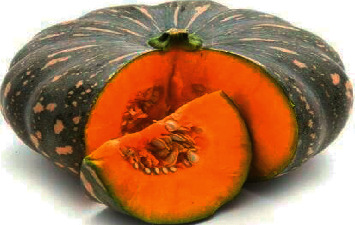
Pumpkin (*Cucurbita maxima*).

Pumpkin is considered as an important vegetable crop due to its nutritional values. It is widely cultivated and consumed throughout the world. The most common species of pumpkin that are grown and used worldwide are *Cucurbita maxima*, *Cucurbita pepo*, and *Cucurbita moschata* [1, 2]. The commonly consumed pumpkin species in Ethiopia is *C. maxima* (Figure 1). In Ethiopia, this vegetable is locally known as “Dubba”. All parts of pumpkins are consumed without producing any waste. Every part of pumpkin are excellent sources of bioactive compounds like carotenoid, vitamins, minerals, fatty acids, carbohydrates, proteins, amino acids, volatile compounds, and phenolic compounds. It is believed that bioactive compounds of pumpkin have a protective role against many diseases, including hypertension, diabetes, and cancer and coronary heart diseases [3–8]. Despite these features, pumpkin is an under-utilized vegetable in Ethiopia.

**Figure 2:**
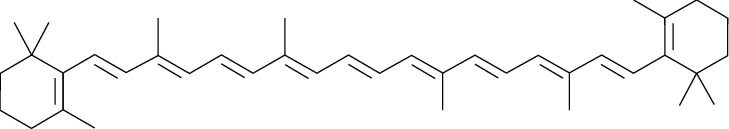
Structure of *β*-carotene.

One of the important components of pumpkin is *β*-carotene (Figure 2). It is a soluble plant pigment. *β*-Carotene is an important functional ingredient among the carotenoid compounds, due to its pro-vitamin A activity and its antioxidant action by scavenging oxygen radicals and reducing oxidative stress in the organism [6]. Consumption of *β*-carotene-rich food has the ability to increase the vitamin A intake among women and children who are always at risk of vitamin A deficiency [5]. In Ethiopia, vitamin A deficiency leads to 80,000 deaths in a year and affects 61% of preschool children [5]. The recommended daily intake of vitamin A is 900 mcg for men, 700 mcg for women, and 300 to 600 mcg for children [8]. Consumption of pumpkin as a vegetable and/or processed food can be a good supplement of vitamin A for Ethiopians.

The *β*-carotene content in vegetables are influenced by many factors such as variety, level of maturity, climatic conditions, geographical location of production, part of the plant utilized, environmental conditions during agricultural production, postharvest handling, processing, storage conditions, and types of solvent used for the extraction. The *β*-carotene content in the pumpkin cultivated in different countries has been determined. However, to the best of our knowledge, there is no reported data on *β*-carotene content in any vegetable, including pumpkin (flesh, peel, and seeds) grown in Ethiopia.

Different analytical methods have been reported in the literature for the determination of *β*-carotene in fruits and vegetables. For quantitative analysis of carotenoids, UV-VIS spectrophotometry is the most convenient method [6, 8–12], by measuring the absorbance at different wavelengths. High-performance liquid chromatographic (HPLC) methods [13–15] were reported for the identification and quantification of *β*-carotene in fruits and vegetables. For carotenoid evaluation, NIR spectroscopy in the wavelength region between 800 and 2500 nm were studied to evaluate *β*-carotene content [12, 16]. The use of infrared spectroscopy in the study of *β*-carotene has also been reported. The development of Fourier transform infrared (FTIR) spectroscopy that operates in the mid-infrared region (4000–400 cm^−1^) has been proven to be a powerful tool for quantitative determination of *β*-carotene [12, 16–19]. Each of these methods has its own advantages and limitations. The researchers are still investigating to develop simple, fast, sensitive, and selective methods for the determination of *β*-carotene in different vegetables. Hence, the objective of the present research work was to evaluate all the analytical parameters to improve the performance of UV-VIS, NIR, and FTIR methods to determine *β*-carotene content in three parts of the pumpkin flesh, peel, and seed samples.

## 2. Materials and Methods

### 2.1. Instruments and Apparatus

Samples were weighed using electronic balance (ARA520, OHAUS CORP, China). Blending device mortar and pestle was used to ground the dried pumpkin samples. Centrifuge machine (800D, China), double-beam spectrophotometer (Lambda 950-UV-Vis-NIR, Perkin Elmer, UK) interfaced with a computer using 2 nm resolution in a 1 cm path length quartz cell and FTIR spectrometer (Perkin Elmer Spectrum 65 Spectrophotometer, USA) with a sample holder of zinc selenide crystal in the attenuated total reflectance (ATR) mode were used for determination of *β*-carotene.

### 2.2. Chemicals

Dichloromethane (ACS ISO-Reagent, European Pharmacopoeia, 99.9%), acetone (ACS ISO-Reagent, European Pharmacopoeia, 99.8%), methanol (Jeulin, France, 99.5%), acetonitrile (Scharlau Chemie SA, Spain, 99.7%), and cyclohexane (Fisher Chemical, UK, 99%) were used for extraction of *β*-carotene. Pumpkin samples were collected from Dukem and Debre Berhan (Ethiopia). *β*-Carotene standard (Sigma-Aldrich, 99%) were obtained from Ethiopian Public Health Institute.

### 2.3. Preparation of *β*-Carotene Standard Solutions for the UV-VIS Method

A 15 µg/mL standard stock solution of *β*-carotene was prepared by dissolving 750 µg of the standard *β*-carotene in 50 mL volumetric flask in acetone. From this stock solution, serial dilutions were made to obtain 0.1, 0.5, 1.5, 3, 6, 9, and 12 µg/mL of *β*-carotene. The working standard solutions were scanned in the spectral range (350–600 nm) selected for this study. The absorption spectral data were collected from their typical absorption peak maximum obtained at 453 nm for plotting the calibration curves.

### 2.4. Preparation of *β*-Carotene Standard Solutions for the NIR Spectroscopic Method

A 250 µg/mL standard stock solution of *β*-carotene was prepared by dissolving 12,500 µg of the standard *β*-carotene in 50 mL volumetric flask in acetone. From this stock solution, serial dilutions were made to obtain 12.5, 20, 35, 60, 100, 160, 190, and 250 µg/mL of *β*-carotene. The working standard solutions were scanned in the spectral range (1000–1600 nm) selected for this study. The absorption spectral data were collected from their typical absorption peak maximum obtained at 1415 nm for plotting the calibration curves.

### 2.5. Preparation of *β*-Carotene Standard Solutions for the FTIR Spectroscopic Method

A 100 µg/mL standard stock solution of *β*-carotene was prepared by dissolving 5000 µg of the standard *β*-carotene in 50 mL volumetric flask in acetonitrile. From this stock solution, serial dilutions were made to obtain 4, 8, 12, 23, 55, and 100 µg/mL of *β*-carotene. About 1 mL of the standard solution was placed in the zinc selenide crystal to fully cover its surface for the spectral measurements. The working standard solutions were scanned in the spectral range (1000–4000 cm^−1^) selected for this study. The absorption spectral data were collected from their typical absorption peak maximum obtained at 1500–2000 cm^−1^ for plotting the calibration curves.

### 2.6. Preparation of Pumpkin Peel, Flesh, and Seed Powder Samples

Pumpkin samples were washed with fresh running water and then with distilled water to remove any foreign materials attached. The separation of the three parts (peel, flesh, and seed) of the pumpkin was done manually with the help of a knife. The three parts of the pumpkin samples were cut in to small pieces and dried at room temperature for 1 week. The dried samples were ground with mortar and pestle. Finally, the powdered samples were used for the extraction of *β*-carotene.

### 2.7. Extractions of *β*-Carotene

*β*-Carotene extraction was done according to the method reported by Barba et al. [11] with some modifications. The *β*-carotene was extracted by soaking 0.5 g of samples for UV-VIS and 5.0 g of samples for NIR and FTIR in 25 mL of different solvents (dichloromethane, acetone, methanol, acetonitrile, and cyclohexane) at room temperature under dark condition in order to get a complete extraction. The mixture was magnetically stirred for 30 min. The extracts were centrifuged to separate the supernatant, and these operations were repeated until the pulp was completely colorless. The volume was made up to 50 mL with the extracting solvents. Finally, the absorbance of the extracts was measured using UV-VIS, NIR, and FTIR methods.

### 2.8. Method Validation

Method validation was evaluated in terms of linearity, accuracy, precision, limits of detection (LOD), and quantitation (LOQ). Linearity of the method was performed in a range of 0.1 to 12 µg/mL for UV-VIS, 12.5 to 250 µg/mL for NIR, and 4 to 100 µg/mL for FTIR methods and correlation coefficients (*R*^2^) were evaluated. Precision was determined from repeatability, intraday, and interday precision, and relative standard deviation (RDS) was calculated. Repeatability was from repetitive UV-VIS, NIR, and FTIR measurement of standard *β*-carotene solution (*n* = 6). Intraday precision was determined on the same day (*n* = 3), and interday precision was determined on different days (*n* = 3). Accuracy was estimated by the standard addition method, and recovery (%*R*) was calculated. Standard *β*-carotene solution was added into the sample, and absorption of the spiked and unspiked samples was measured. The recovery (%*R*) was calculated using the formula (amount found/amount added) × 100. LOD and LOQ were calculated from (3 × SD)/*s* and (10 × SD)/*s*, where SD and *s* were standard deviation of blank measurement (*n* = 6) and slope of calibration curve, respectively.

## 3. Results and Discussion

### 3.1. Extraction Solvent Optimization

**Figure 3:**
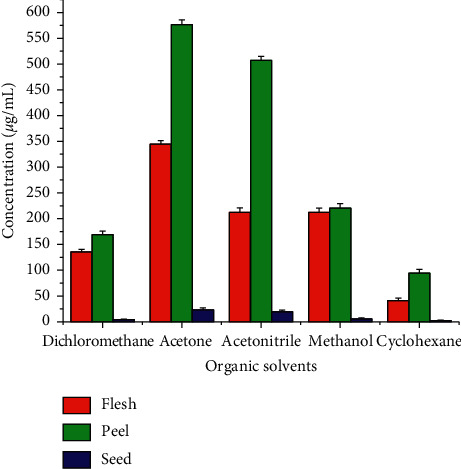
Results of efficiency of five extraction solvents (dichloromethane, acetone, methanol, acetonitrile, and cyclohexane) in pumpkin flesh, peel, and seed samples based on UV-VIS absorption analysis.

To obtain the best extraction efficiency, different extraction solvents were optimized. Five extraction solvents (dichloromethane, acetone, methanol, acetonitrile, and cyclohexane) were used to compare the extraction efficiency of *β*-carotene from pumpkin flesh, peel, and seed. Better extraction efficiency of *β*-carotene was observed when using acetone than the other extraction solvents, as determined by UV-VIS absorption analysis. Cyclohexane extraction resulted in lower extraction efficiency than the other extraction solvents (Figure 3). Therefore, acetone was selected as a solvent for the extraction of *β*-carotene for further analysis in this study.

### 3.2. Determination of *β*-Carotene in Pumpkin Peel, Flesh, and Seed Using the UV-VIS Method

#### 3.2.1. Identification

**Figure 4:**
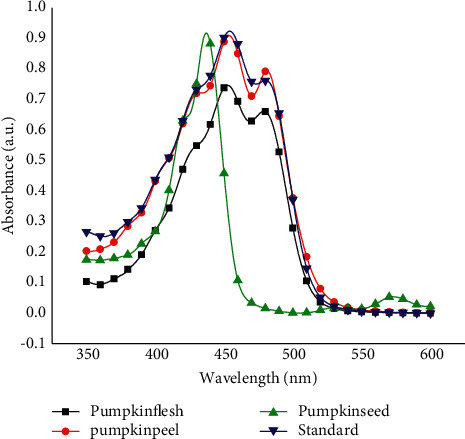
UV-VIS spectra of standard *β*-carotene and *β*-carotene extracts from pumpkin flesh, peel, and seed samples.

The UV-VIS spectrum of *β*-carotene in acetone was scanned from 350 to 600 nm, and maximum absorption was obtained at 453 nm. This was in good agreement with that reported in literature [6, 8, 11–13]. The UV-VIS spectra of pumpkin flesh and peel extracts showed the maximum UV-VIS absorption at the same wavelength (Figure 4). This confirmed that the extraction procedure was valid and the extract contained *β*-carotene. In the case of pumpkin seed, the extract showed the maximum UV-VIS absorption at 440 nm (Figure 4). The shift in absorption maximum and curve shape may be attributed to the different chemical environment in the seed as compared to the flesh and peel. The good selectivity of the newly developed UV-VIS method is evidenced by an overlapping standard and sample spectra, which clearly indicates the reduction of the matrix interference to a negligible levels.

#### 3.2.2. Method Validation for UV-VIS

**Figure 5:**
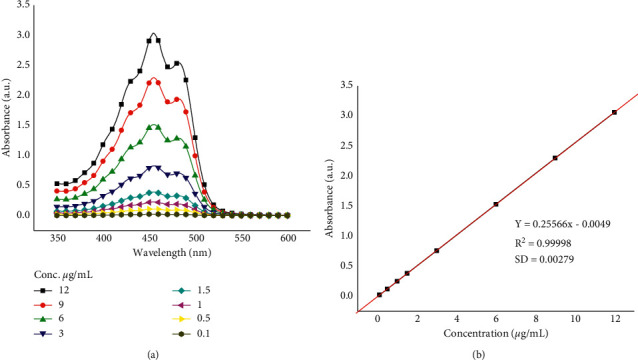
UV-VIS spectra of different concentrations of standard *β*-carotene in acetone (a) and calibration curve (b).

Linearity of the method was performed in a range of 0.1 to 12 µg/mL, and the method showed good linearity with a regression of *y* = 0.25566*x*–0.0049 (*R*^2^ = 0.999) (Figure 5). In addition, both the detection limit and quantification limit were calculated to be 0.034 µg/mL and 0.1 µg/mL, respectively. Method repeatability showed RSD% of 1.5%. Intraday and interday precision were RSD 1.8% to 2.8% and 3.7% to 11%, respectively. Method accuracy was evaluated by standard addition to the sample, and the results obtained showed good percent recovery (%*R*) of 83% to 93%.

#### 3.2.3. UV-VIS Method for Real Sample Analysis

The developed UV-VIS method was applied for the determination of *β*-carotene content in the acetone extract of the pumpkin peel, flesh, and seeds samples. *β*-Carotene content in the sample (µg/g) was calculated using calibration curve. The results are given in Table 1. Content of *β*-carotene was obtained higher amount in pumpkin peel followed by pumpkin flesh and pumpkin seed samples (Table 1).

### 3.3. Determination of *β*-Carotene in Pumpkin Flesh, Peel, and Seed Samples Using the NIR Spectroscopic Method

#### 3.3.1. Identification

**Figure 6:**
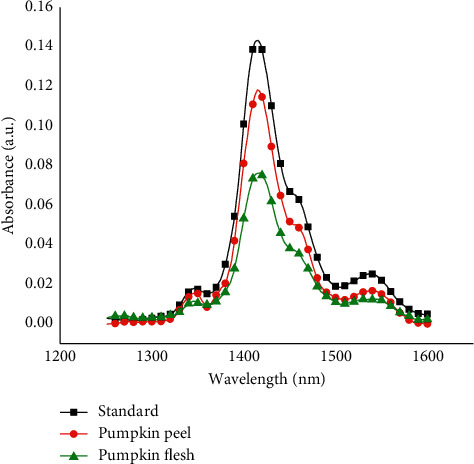
NIR spectra of standard *β*-carotene and *β*-carotene from pumpkin flesh and peel with acetone extracts.

The NIR spectra of *β*-carotene in acetone were scanned from 1000 to 1600 nm spectral range, and maximum absorption was obtained at 1415 nm, which is attributed to the first overtone band of C–H stretching mode. This was in good agreement with that reported by Nokkaew et al. and Rungpichayapichet et al. [20, 21]. NIR spectra of pumpkin flesh and peel extract revealed maximum NIR absorption at the same wavelength, that is, at 1415 nm (Figure 6). This confirmed that the extraction procedure was valid and the extract contained *β*-carotene. The perfectly overlapping standard and sample spectra clearly indicate the reduction of the matrix interference to a negligible levels. Furthermore, this also demonstrates the excellent selectivity of the newly developed NIR method.

#### 3.3.2. Method Validation for NIR Spectroscopy

**Figure 7:**
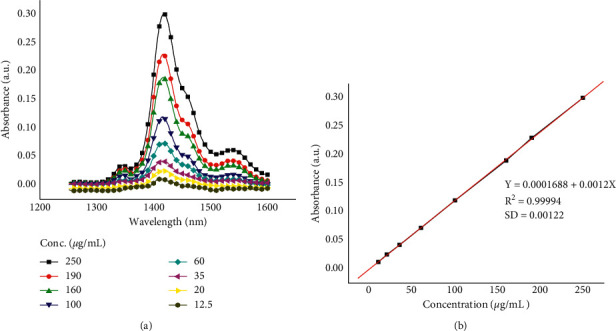
NIR spectra of different standard concentrations of *β*-carotene (a) and calibration curve (b).

Linearity of the method was performed in concentration range between 12.5 and 250 µg/mL, and the method showed good linearity with regression equation of *y* = 0.0001688 + 0.0012*x* and regression coefficient of *R*^2^ = 0.99994 (Figure 7). In addition, both detection limit and quantification limit were calculated to be 3.4 µg/mL and 10 µg/mL, respectively. Method repeatability showed RSD of 3.7%. Intraday and interday precision were RSD in ranges of 1.8% to 3.5% and 4.9% to 11%, respectively. Method accuracy was evaluated by standard addition to the sample, and the results obtained showed good percent recovery (%*R*) of 92.0% to 96.0%.

#### 3.3.3. NIR Spectroscopic Method for Real Sample Analysis

The content of *β*-carotene in pumpkin (flesh, peel, and seed) samples was determined by NIR spectroscopic method. The content of *β*-carotene (µg/g) was calculated using the calibration curve. Content of *β*-carotene was obtained higher in pumpkin peel, followed by pumpkin flesh. The *β*-carotene content in pumpkin seed was not detected in NIR spectroscopic method (Table 2).

### 3.4. Determination of *β*-Carotene Content in Pumpkin Flesh, Peel, and Seed Samples Using the FTIR Spectroscopic Method

#### 3.4.1. Identification

**Figure 8:**
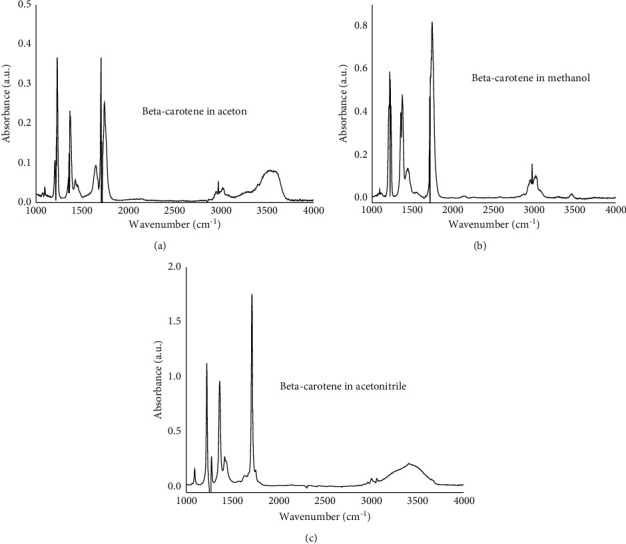
FTIR-ATR spectra of *β*-carotene in acetone (a), methanol (b), and acetonitrile (c).

New FTIR method was developed for quantitation of *β*-carotene in pumpkin peel, flesh, and seed samples using a liquid sampling technique. In order to choose an appropriate solvent for the analysis, pure *β*-carotene was dissolved in three different solvents (acetone, methanol, and acetonitrile) and FTIR spectra were recorded (Figure 8).

The absorption bands from 2900 to 3050 cm^−1^ were the common asymmetric and symmetric stretching modes of the C–H groups. The weak absorption band observed at 3005 cm^−1^ is due to the trans –CH = CH of the *β*-carotene. The sharp band at 1462 cm^−1^ arose from the asymmetric deformation mode, whereas the band at 1360 cm^−1^ was the symmetric deformation mode of the C–H group. The peak around 1500 to 2000 cm^−1^ corresponds to C = C double bond stretching vibrations of *β*-carotene. For quantitative analysis, the intensities in the spectral region 1500 to 2000 cm^–1^ were chosen because the peak at 1710 cm^–1^ have strong intensity in the pure *β*-carotene as well as in the given samples, and thus the wavenumber (cm^–1^) 1710 cm^–1^ was chosen for the quantification propose. However, *β*-carotene spectra in acetone and methanol solvents indicated some interfering absorption bands of the solvent itself at around 1710 cm^–1^. Due to this reason, acetonitrile was selected to be an appropriate solvent. FTIR spectrum of *β*-carotene in acetonitrile was scanned from 1500 to 2000 cm^–1^, and maximum absorption was obtained at 1710 cm^–1^ which is due to the C = C stretching vibrational mode.

**Figure 9:**
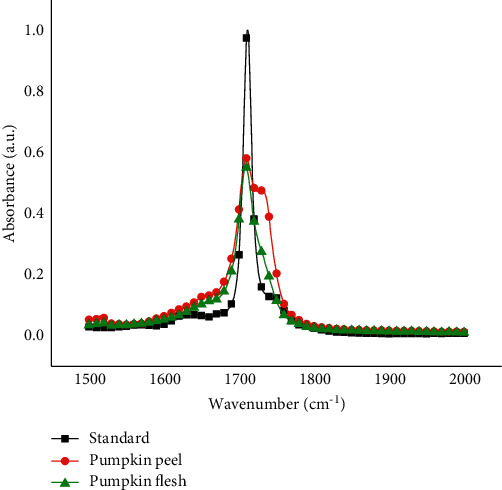
FTIR spectra of standard *β*-carotene and *β*-carotene from pumpkin flesh and peel in acetonitrile extracts.

Subsequently, FTIR spectra of pumpkin flesh and peel extracts were recorded and showed maximum absorption at the same wavenumber (Figure 9). This confirmed that the extraction procedure was valid and the extract contained *β*-carotene. The overlapping of standard and sample spectra clearly indicates the reduction of the matrix interference to a negligible level. Furthermore, this also demonstrates the good selectivity of the newly developed FTIR method.

#### 3.4.2. Method Validation for FTIR Spectroscopy

**Figure 10:**
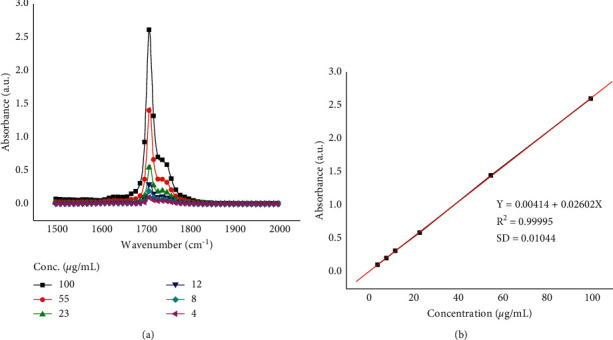
FTIR spectra of different concentrations of standard *β*-carotene in acetonitrile (a) and calibration plot (b).

Linearity of the method was performed in a concentration range between 4 and 100 µg/mL. The method showed good linearity with regression equation of *y* = 0.00414 + 0.02602*x* and regression coefficient *R*^2^ = 0.99995 (Figure 10). In addition, both detection limit and quantification limit were 1.3 µg/mL and 3.9 µg/mL, respectively. Method repeatability showed RSD of 0.95%. Intraday and interday precision revealed RSD of 0.69% to 5.6% and 3.9% to 10%, respectively. Method accuracy was evaluated by standard addition to the sample and the results obtained showed good percent recovery (%*R*) of 95% to 96%.

#### 3.4.3. FTIR Method for Real Sample Analysis

The content of *β*-carotene in the pumpkin flesh, peel, and seed sample was determined by FTIR spectroscopic method. The *β*-carotene content (µg/g) was calculated using calibration curve. Content of *β*-carotene was obtained higher in pumpkin peel, followed by pumpkin flesh. The *β*-carotene content in pumpkin seed was not detected using FTIR spectroscopic method (Table 3).

### 3.5. Comparison of Results Obtained by Three Developed Methods for *β*-Carotene Determination

In this study, three different methods were developed for the quantitative determination of *β*-carotene in the pumpkin flesh, peel, and seed by using acetone and acetonitrile as the extraction solvents. The analytical parameters such as correlation coefficient (*R*^2^), linear range, limit of detection (LOD), limit of quantification (LOQ), and relative standard deviation (RSD) obtained by the newly developed UV-VIS method are in good agreement with results reported by Karnjanawipagul et al. [7], and analytical parameters obtained in this study are better than results reported by Biswas et al. [22] (Table 4). Some of the analytical parameters such as limit of detection (LOD) and limit of quantification (LOQ) of the method were not reported by Biswas et al. [22].

The analytical parameters for NIR and FTIR methods were not reported except correlation coefficient (*R*^2^) in the literature methods for NIR [23, 24] and for FTIR methods [16, 25].

Since, the objective of the present research work was to improve the performance of analytical methods (UV-VIS, NIR, and FTIR) to determine *β*-carotene content in three parts of pumpkin flesh, peel, and seed samples, all the analytical parameters such as correlation coefficient (*R*^2^), linear range, limit of detection (LOD), limit of quantification (LOQ), recoveries (%*R*), and relative standard deviation (%RSD) have been evaluated and reported for the newly developed (UV-VIS, NIR, and FTIR) methods. The three newly developed methods are applicable to determine *β*-carotene with good linearity, precision, accuracy, and sensitivity in the three parts of pumpkin. Thus, the developed methods indicate better performance than previously published methods (Table 4).

Some of these RSD% values were slightly higher because *β*-carotene was unstable and easily degraded at room temperature. However, they were in acceptable ranges according to AOAC [26] regulation. Validation data indicated that the developed methods showed good linearity, precision, accuracy, and sensitivity, which could be used for the determination of *β*-carotene in pumpkin flesh, peel, and seed samples.

### 3.6. Comparison of Results Obtained by the Present Developed Methods with Literature-Reported Methods

In this study, *β*-carotene content was obtained in the range of 317 to 341, 340 to 445, and 12 to 17 µg/g in pumpkin flesh, peel, and seed using UV-VIS method; 289 to 313 and 376 to 451 µg/g in pumpkin flesh and peel using NIR method; 102 to 148 and 210 to 287 µg/g in pumpkin flesh and peel using FTIR method. *β*-Carotene was not detected in pumpkin seed using NIR and FTIR methods.

To further validate the developed methods, it is necessary to compare the results with literature-reported methods such as UV-VIS, NIR, FTIR, and HPLC. There are different reported ranges of *β*-carotene content of different vegetables including pumpkin flesh, peel, and seed. In a study with pumpkin (*C. moschata*) flesh, *β*-carotene content varied between 168 and 202 µg/g [10]. Carotenoids determination in pumpkin flesh content varied between 156 and 2137 µg/g [16]. The investigation performed in crude palm oil reported in the range of 260 to 783 µg/g [12].

In the investigation performed in pumpkin (*C. maxima*) flesh and peel using HPLC-DAD method, *β*-carotene content ranged from 17 to 263 µg/g in flesh and 10 to 403 µg/g in peel [15]. Kim et al. [27] reported the determination of different carotenoids in pumpkin (*C. maxima*) flesh, peel, and seed, the content of *β*-carotene were obtained 17 µg/g in flesh, 123 µg/g in peel, and 31 µg/g in seed powder samples. Pongjanta et al. [28] prepared pumpkin powder to utilize it in different bakery products and made a comparison of *β*-carotene in fresh pumpkin and pumpkin powder. The amount of *β*-carotene reported in fresh and powder pumpkin were 24 µg/g and 73 µg/g, respectively. Nakazibwe et al. [29] also reported *β*-carotene content 27 to 1215 *μ*g/g in three variety of pumpkin flesh samples (Table 5).

In this study, *β*-carotene content was determined in the three parts of pumpkin namely flesh, peel, and seed, collected from two different sample area (Dukem and Debre Berhan) of Ethiopia. The variation in *β*-carotene content observed in the three newly developed methods may be attributed to the difference in the origin and ripping stages of the pumpkin samples. Therefore, to make the comparison meaningful, we have conducted the experiments on the same pumpkin samples by the FTIR-ATR and UV-VIS methods, and the results obtained by the two newly developed methods are in good agreement. The results are given in Table 6.

We have conducted further experiment to determine *β*-carotene content in the pumpkin flesh by a literature-reported UV/VIS spectrophotometric method [30]. The results obtained on the *β*-carotene content in the pumpkin flesh by the newly developed UV/VIS spectrophotometric method are comparable with the literature-reported method [30]. The results are given in Table 6, which demonstrate the validation of the newly developed method.

The variation in the content of *β*-carotene in three parts of pumpkin flesh, peel, and seed may be due to the presence of different chemical compositions. Kim et al. [27] reported that *β*-carotene concentration in three species of pumpkin such as *C. pepo*, *C. moschata*, and *C. maxima* are found to be high in peels compared to flesh and seeds. Generally, results obtained in this study are comparable with the results literature reported using UV-VIS spectrophotometry [10], FTIR [12, 16], and HPLC [15, 27–29] methods (Table 5). It should be noted that the pumpkin sample used in the FTIR-ATR method was different from that used in the UV-VIS and NIR methods. As the *β*-carotene content in the pumpkin sample depends on the origin and maturity of the fruit and as two different fruits were used for the FTIR-ATR method and UV-VIS and NIR methods, the results of *β*-carotene content in the two pumpkin samples were different. To make the comparison meaning, we have conducted the experiments on the same pumpkin samples, and the results obtained by the three newly developed methods are in good agreement. The new results are included in Table 6.

## 4. Conclusion

This study evaluated all the analytical parameters and demonstrated improvement in the performance of the analytical methods for the determination of *β*-carotene in pumpkin flesh, peel, and seed samples using UV-VIS, NIR, and FTIR methods. All the three methods are applicable to determine *β*-carotene content in pumpkin with good linearity, precision, accuracy, and sensitivity. The content of *β*-carotene in pumpkin flesh, peel, and seed are comparable with those reported in literature in vegetables including pumpkin. Varied amounts of *β*-carotene in pumpkin samples were due to several environmental factors (nutrient, water, and soil), age, parts of pumpkin, and species of pumpkin.

## Figures and Tables

**Table 1:** Content (*µ*g/g) of *β*-carotene from the three parts of pumpkin, namely, peel, flesh, and seed samples using the UV-VIS method.

Sample area	*β*-Carotene in pumpkin (*µ*g/g)		
	Flesh	Peel	Seed
Dukem	341	445	17
Debre Berhan	317	340	12

**Table 2:** Content (*µ*g/g) of *β*-carotene from the three parts of pumpkin (peel, flesh, and seed) samples using the NIR spectroscopic method.

Sample area	*β*-Carotene in pumpkin (*µ*g/g)		
	Flesh	Peel	Seed
Dukem	313	451	ND
Debre Berhan	289	376	ND

ND = not detected.

**Table 3:** Content (*µ*g/g) of *β*-carotene in the pumpkin peel, flesh, and seed samples using the FTIR spectroscopic method.

Sample area	*β*-Carotene in pumpkin (*µ*g/g)		
	Flesh	Peel	Seed
Dukem	210	287	ND
Debre Berhan	102	148	ND

ND = not detected.

**Table 4:** Analytical parameters of the proposed methods such as *R*^2^, LODs, LOQs, RSDs, and recoveries compared with the previously published methods.

Methods	Linearity range (*µ*g/mL)	*R* ^2^	LOD (*µ*g/mL)	LOQ (*µ*g/mL)	RSD (%)	Recovery (%)	Reference
UV-VIS	0.1–12.0	0.999	0.034	0.10	1.5–11	83–93	This study
UV-VIS	1–8	0.999	0.04	0.11	6.4	100	[7]
UV-VIS	0.015–8	0.994	NR	NR	3.4–8.9	67.8–98.8	[22]
NIR	12.5–250	0.999	3.400	10.2	1.8–11	92–96	This study
NIR	0–24	0.81	NR	NR	NR	NR	[23]
NIR	2.3–28	0.81	NR	NR	NR	NR	[24]
FTIR	4–100	0.999	1.320	3.97	0.7–10	95–96	This study
FTIR	1.8–6.6	0.91	NR	NR	NR	NR	[25]
FTIR	183–2137	0.95	NR	NR	NR	NR	[16]

NR = not reported.

**Table 5:** Comparison of *β*-carotene content in the pumpkin flesh, peel, and seed samples obtained by three newly developed methods with the values reported in the literature methods.

Methods	*β*-Carotene in pumpkin (*µ*g/g)			Reference
	Flesh	Peel	Seed	
UV-VIS	317–341	340–445	12–17	This study
NIR	289–313	376–451	ND	This study
FTIR	102–210	148–287	ND	This study
UV-VIS	168–202	—	—	[10]
FTIR	156–2137	—	—	[16]
HPLC	17–263	10–403	—	[15]
HPLC	31	123	17	[27]
HPLC	27–1215	—	—	[29]

**Table 6:** *β*-Carotene content (*µ*g/g) of the same pumpkin flesh using different methods.

Comparison of results of the two newly developed UV-VIS and FTIR methods	

UV-VIS	FTIR

202 *µ*g/g	198 *µ*g/g

Comparison of results of the newly developed method with published method [30]	

Present UV-VIS	UV-VIS published method [30]

202 *µ*g/g	204 *µ*g/g

## Data Availability

All the data are included within the manuscript.
